# DNM1 Mutation in a child associated with progressive bilateral mesial temporal sclerosis

**DOI:** 10.1002/ccr3.1793

**Published:** 2018-09-12

**Authors:** Alexandra Lazzara, Sheila Asghar, Thomas Zacharia, Debra Byler

**Affiliations:** ^1^ Penn State Health Milton S. Hershey Medical Center Hershey Pennsylvania

**Keywords:** genetics, neurology, neurosurgery, pediatrics and adolescent medicine

## Abstract

This case represents a novel association of a DNM1 gene mutation with status epilepticus and progressive bilateral mesial temporal sclerosis. This could have future implications for treatment in patients with DNM1 mutation and refractory epilepsy as the mesial temporal sclerosis may become bilateral, making the patient a poor surgical candidate.

## 1 BACKGROUND

This case report represents a unique presentation of a DNM1 gene mutation. Dynamin‐1 is required for synaptic vesicle recycling.^1^ Heterozygous mutations in DNM1 have been associated with early infantile epileptic encephalopathy. Cases have described patients with severe intellectual disability, lack of speech, hypotonia, and inability to walk.^1^ Patients with de novo mutations in the DNM1 gene have also been reported to develop infantile spasms and Lennox‐Gastaut Syndrome.^1^ Other case studies have reported developmental regression, autism spectrum disorders, cortical visual impairment, behavioral concerns, and macrocephaly. A recent study by Von Spiczak et al^2^ reported prominent hyperkinetic movements, dystonic posturing of the head and limbs, and/or ataxic gait in patients who achieved independent ambulation in patients with this variant.

These mutations typically arise de novo, but cases have been reported with inheritance from a parent with somatic mosaicism.^1^ Li et al found that dynamin 1 was significantly higher in patients with temporal lobe epilepsy. Inhibiting dynamin 1 also increased the latency time of the first seizure and decreased the frequency and severity of the seizures, suggesting that altering levels of dynamin 1 may contribute to the development of epileptic seizures.^3^ Zhang et al found that phosphor‐dynamin‐1 is downregulated in the hippocampus of children and rats with mesial temporal lobe epilepsy during seizures suggesting involvement of phosphorylation or dephosphorylation of dynamin‐1 in the development of mesial temporal lobe epilepsy.^4^

## 2 CASE HISTORY

This is a 12‐year‐old male with intellectual disability and significant motor delay. He did not walk until 3 years old and began talking at 1.5 years. He had neonatal seizures at 5 days of age which were treated with phenobarbital until 5 months of age. He had hypotonia and was diagnosed with Autism Spectrum Disorder (ASD). At 7 years of age, he was diagnosed with focal epilepsy with impaired awareness after two convulsive seizures and a prolonged postictal period lasting 7‐8 hours. A thorough workup including EEG, MRI, laboratory studies, and toxicology demonstrated increased signal in the left hippocampus. He was started on oxcarbazepine and was seizure free until he was hospitalized 10 months later in status epilepticus requiring intubation. Because of recurrent seizures, trials of maximal doses of levetiracetam and oxcarbazepine were employed without success. Valproic acid was later added. Trials of lamotrigine and topiramate were discontinued due to tics and cognitive slowing, respectively. A referral to a comprehensive epilepsy center resulted in a diagnosis of refractory epilepsy without recommendation for epilepsy surgery.

**Figure 1:**
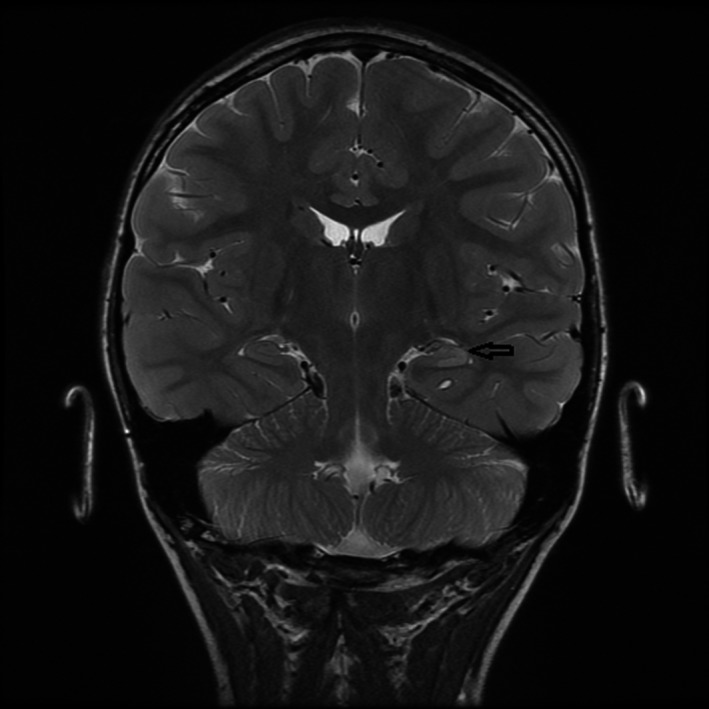
Epilepsy protocol MRI brain through hippocampi at baseline: Coronal T2 image reveal hyperintense signal in the left hippocampus (arrow). Right hippocampus is normal in signal and morphology

**Figure 2:**
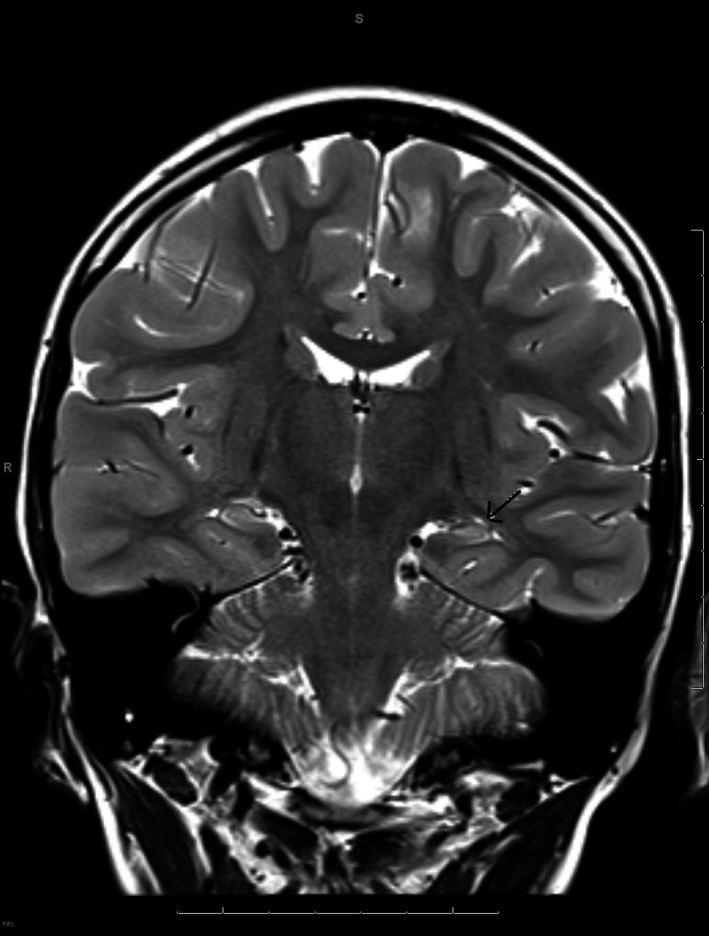
Epilepsy protocol MRI brain through hippocampi after 4 y: Coronal T2 sequence through hippocampi demonstrate a “layered” pattern of alternating T2 hyperintensity and isointensity involving the head and body of hippocampus(arrow)

Serial EEGs showed mild diffuse slowing and multifocal epileptiform activity. His MRI at the time of diagnosis showed increased T2 signal in the left mesial temporal region involving head and body of hippocampus (Figure 1). There was no volume loss of collateral white matter and fornix. The right hippocampus was normal. A repeat MRI 4 years later showed increased T2 signal and loss of normal morphology of hippocampus bilaterally. There was prominence of choroid fissure bilaterally. Findings were better demarcated on the left and new on the right compared to prior MRI. There was a distinct pattern of alternating T2 hyperintensity and isointensity involving the head and body of hippocampus (Figure 2). He was referred for possible surgical intervention but it was determined that surgery was not appropriate at this time. Genetic testing revealed that the patient is heterozygous for a variant of the DNM1 gene specifically c.796C.T(p.Arg266Cys). According to Von Spiczak et al (2017), the most common mutation in a cohort of 21 patients with DNM1 encephalopathy was the p.ARG237Trp mutation. Parental testing was requested but never obtained by the parents.

## 3 DISCUSSION

Mesial temporal sclerosis (MTS) has been associated with mutations in the SCN1A gene and febrile status epilepticus.^5^ However, it has not yet been reported in association with a DNM1 gene mutation. Additionally, this patient's mesial temporal sclerosis developed over time. Initially, it was very mild and only on the left side, but over the course of 3 years, the severity increased on the left with involvement of the right. The role of recurrent status epilepticus in the eventual development of mesial temporal sclerosis is unclear, and we cannot completely rule out a contribution of the natural history in this case. However, the underlying mutation is likely the driving factor in the patient's progression over time. There is no history to suggest association with a febrile illness, and FIRES is thought to be unlikely in this clinical setting.

More commonly, patients with MTS tend to have focal seizures with impairment of consciousness rather than secondarily generalized seizures. Some authors have indicated that status epilepticus in patients with mesial temporal sclerosis is not common.^6^ Interestingly, our patient presented with mostly generalized tonic‐clonic seizures which progressed to status epilepticus. Although focal onset was not observed, these were most likely focal onset with bihemispheric spread. Moreover, the imaging pattern of “alternating T2 hyperintensity and isointensity” or “layered signal intensity” (Figure 2) on coronal T2 weighted images was distinct and not reported in imaging literature of mesial temporal sclerosis.

## 4 CONCLUSION

This case represents a novel association of a DNM1 gene mutation with status epilepticus and progressive bilateral mesial temporal sclerosis. This could have future implications for patients identified to have mesial temporal sclerosis. If they are found to have the DNM1 gene mutation, the mesial temporal sclerosis may progress and become bilateral, making the patient a poor surgical candidate. Identifying this mutation may help to guide treatment in patients with mesial temporal sclerosis.

## CONFLICT OF INTEREST

None declared.

## AUTHORSHIP

AL: gathered materials for submission, drafted the initial version of the manuscript, incorporated edits and revisions, and submitted the final version of the manuscript. SA and DB: reviewed the literature, contributed their expertise in the field of pediatric neurology and provided revisions and edits leading to the final version of the manuscript. TZ: contributed content and expertise in the field of neuroradiology and provided the images along with annotations.
